# Hyponatriämie: Ätiologie, Diagnostik und Akuttherapie

**DOI:** 10.1007/s00063-023-01049-0

**Published:** 2023-08-30

**Authors:** Fabian Perschinka, Paul Köglberger, Sebastian J. Klein, Michael Joannidis

**Affiliations:** 1 Gemeinsame Einrichtung Internistische Intensiv- und Notfallmedizin, Department für Innere Medizin, Medizinische Universität Innsbruck, Anichstraße 35, 6020 Innsbruck, Österreich; 2 Institut für Anästhesiologie und Intensivmedizin, Klinikum Wels, Grieskirchnerstraße 42, 4600 Wels, Österreich

**Keywords:** Wasser-Elektrolyt-Ungleichgewicht, Syndrom der inadäquaten ADH-Sekretion, Wasservergiftung, Demyelinisierungssyndrom, Workflow, Water-electrolyte imbalance, Inappropriate ADH syndrome, Water intoxication, Osmotic demyelination syndrome, Workflow

## Abstract

Die Hyponatriämie ist eine der häufigsten Elektrolytstörungen in Notaufnahmen und bei hospitalisierten Patient*innen. Die Serum-Natriumkonzentration wird über die Osmoregulation sowie die Volumenregulation kontrolliert. Beides erfolgt über die Freisetzung von antidiuretischem Hormon (ADH). Die inadäquate ADH-Freisetzung (SIADH) kann durch Tumore, Pneumonien, Medikamenteneinnahme oder Drogenkonsum getriggert werden. Auch übermäßige Flüssigkeitsaufnahme kann zur Abnahme der Serum-Natriumkonzentration führen. Rasche Veränderungen in der Serum-Natriumkonzentration führen zu Zellschwellung bzw. Zellschrumpfung, was sich vor allem in neurologischen Symptomen widerspiegelt. Entscheidend ist hierbei die Geschwindigkeit, mit welcher die Hyponatriämie eintritt, und wie lange die Hyponatriämie schon besteht. In der Diagnostik der Hyponatriämie sind neben der laborchemischen Bestimmung auch eine klinische Untersuchung sowie Harnanalysen von zentraler Bedeutung.

## Abstract (en)

Hyponatremia is one of the most common electrolyte disorders in emergency departments and hospitalized patients. Serum sodium concentration is controlled by osmoregulation and volume regulation. Both pathways are regulated via the release of antidiuretic hormone (ADH). Syndrome of inappropriate release of ADH (SIADH) may be caused by neoplasms or pneumonia but may also be triggered by drug use or drug abuse. Excessive fluid intake may also result in a decrease in serum sodium concentration. Rapid alteration in serum sodium concentration leads to cell swelling or cell shrinkage, which primarily causes neurological symptoms. The dynamics of development of hyponatremia and its duration are crucial. In addition to blood testing, a clinical examination and urine analysis are essential in the differential diagnosis of hyponatremia.

## Lernziele

Nach Lektüre dieses Beitrags …

- verstehen Sie die Grundlagen der Pathogenese und die Ätiologie der Hyponatriämie,
- kennen Sie die therapeutische Bedeutung der Differenzierung in akute und chronische Hyponatriämie gemäß der europäischen Leitlinie,
- sind Sie mit der diagnostischen und differenzialdiagnostischen Herangehensweise bei akuter Hyponatriämie vertraut,
- wissen Sie, wie man im Notfall eine akute Hyponatriämie erkennt und behandelt,
- haben Sie Kenntnis über die weiterführende Therapie nach Linderung der akuten Symptomatik,
- kennen Sie die klinisch relevanten Fallstricke bei der Interpretation der Laborergebnisse und der nachfolgenden Therapie,
- wissen Sie, welche Maßnahmen bei einer Überkorrektur zu ergreifen sind.

## Häufigkeit

Die Hyponatriämie stellt eine in allen Fachbereichen vorkommende **Elektrolytstörung**

Elektrolytstörung

dar, welche häufig als Zufallsbefund festgestellt wird, doch in manchen Fällen auch für ausgeprägte Symptome verantwortlich ist. Während eine Hyponatriämie in den Notaufnahmen in 10 % der Laborbefunde von notfallmäßigen Krankenhausaufnahmen aufscheint [1], liegt sie bei beinahe 20 % der Patient*innen auf einer Intensivstation [2] und bei 30–40 % der Hospitalisierten vor [3, 4, 5].

### Merke

Hyponatriämie tritt in den Notaufnahmen in 10 %, auf Intensivstation in fast 20 % und bei 30–40 % der Hospitalisierten auf.

### Fallbeispiel

Eine 27-jährige Patientin wird in die Notaufnahme eingeliefert. Einweisungsgrund ist ein **synkopales Ereignis**

Synkopales Ereignis

mit anschließendem Krampfgeschehen. Die Patientin hatte fremdanamnestisch am Vorabend und frühmorgens MDMA (3,4-Methylendioxymethylamphetamin, Ecstasy) gemeinsam mit Alkohol konsumiert. In der Notaufnahme präsentiert sich die Patientin (Körpergewicht, KG: 56 kg) kreislaufstabil, normofrequent, nicht mehr kontaktfähig, jedoch noch mit intakten Schutzreflexen. Sie wird auf die Intensivstation aufgenommen. Aufgrund der Symptomatik und der Befunde des Aufnahmelabors wurden initial 100 ml einer 3%igen NaCl-Lösung verabreicht.

*Aufnahmelabor*

- Serum-Natrium: 122 mmol/l
- Serum-Osmolalität: 257 mosmol/kg
- Harn-Osmolalität: 346 mosmol/kg
- Harn-Natrium: 54 mmol/l
- Serum-Ethanol: 0,13 g/l
- Amphetamine im Harn: > 3000

Aufgrund persistierender Symptomatik des zentralen Nervensystems (ZNS) mit **neuerlichem Krampfgeschehen**

Neuerliches Krampfgeschehen

trotz Anstieg der Serum-Natriumkonzentration auf 124 mmol/l erfolgte konsekutiv die Gabe von weiteren 200 ml 3 % NaCl, mit welchen ein Anstieg auf 128 mmol/l erzielt wurde. Bei deutlich gebesserter Symptomatik erfolgte nun keine weitere Natriumsubstitution. Dann wird 4 h später ein Serum-Natrium von 132 mmol/l gemessen. Auffällig ist eine spontane Diurese von 200 ml/h mit wasserklarem Harn.

Was ist die zugrunde liegende Ätiologie dieser schweren Hyponatriämie? Wie erklärt sich der rapide Natriumanstieg ohne Substitution? Welche Gegenmaßnahmen sind zu ergreifen, um einen unkontrollierten Anstieg des Serum-Natriums zu verhindern?

## Definition

Im Allgemeinen hat man sich darauf geeinigt, Natriumwerte im Serum < 135 mmol/l als pathologisch anzusehen. Die europäischen Leitlinien unterteilen auf Basis der **Laborwerte**

Laborwerte

in leichte (135−130 mmol/l), moderate (129 − 125 mmol/l) und profunde (< 125 mmol/l) Hyponatriämie. Der Begriff „schwer“ wird in der Definition bewusst nicht verwendet und der Einteilung der **klinischen Symptomatik**

Klinische Symptomatik

vorbehalten. Diese wird nach Ausprägungsformen in „schwer“, „mittelschwer“ und „leicht“ eingeteilt (Tab. 1; [6]).

**Table 1:** 

Schweregrad	Symptom
Mittelschwer	Übelkeit ohne Erbrechen
	Leichte bis mittelschwere Bewusstseinsstörung
	Kopfschmerzen
Schwer	Erbrechen
	Kardiorespiratorische Dekompensation
	Abnormale und tiefe Schläfrigkeit
	Tonisch-klonische Krampfanfälle
	Koma (Glasgow Coma Scale ≤ 8)

## Diagnostik

In der Labordiagnostik der Serum-Natriumkonzentration kommen **ionenselektive Elektroden**

Ionenselektive Elektroden

zum Einsatz. Beim Verwenden der indirekten Potentiometrie (Standardlabor) als Messmethode kommt es bei Vorliegen hoher Konzentration von Proteinen (multiples Myelom, M. Waldenström) [7, 8] oder Lipiden (Hypertriglyzeridämie) im Blut aufgrund der Probenaufbereitung (Verdünnung) zu falschen Ergebnissen. Zum Ausschluss dieser sog. **Pseudohyponatriämie**

Pseudohyponatriämie

kann die Osmolalität im Serum bestimmt werden, wobei eine iso- oder hypertone Osmolalität bei stark erniedrigter Serum-Natriumkonzentration auf eine Pseudohyponatriämie hinweist. Alternativ kann die Messung mittels Blutgasanalyse-Geräten direkt aus dem Blut erfolgen. Die darin zur Anwendung kommende direkte Potentiometrie misst das Serum-Natrium auch bei Hypertriglyzeridämie oder Hyperproteinämie korrekt [9].

### Merke

Durch die Probenaufbereitung im Labor kann es bei erhöhten Proteinen und Lipiden zur Messung falsch-niedriger Serum-Natriumkonzentrationen kommen.

## Physiologie und Pathophysiologie

### Physiologische Aspekte

Grundlegend gilt es bei der Interpretation vom Serum-Natrium zu verstehen, dass es sich dabei stets um das Verhältnis zwischen der absolut gelösten Menge an Natrium und dem **extrazellulären Wasser**

**Figure 1:**
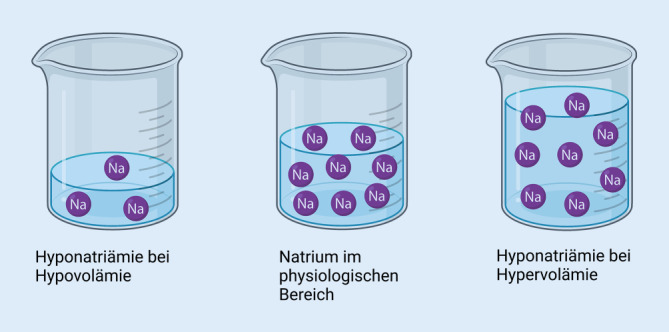

Extrazelluläres Wasser

handelt. Dieselbe absolute Menge an Natrium kann bei Zunahme des extrazellulären Volumens zu einer Hypo- und bei Abnahme desselben zu einer Hypernatriämie führen (Abb. 1). Aufgrund des **dominierenden Einflusses**

Dominierender Einfluss

von Natrium im Extrazellulärraum haben Schwankungen in der Natriumkonzentration Auswirkungen auf die Serum-Osmolalität [10]. Die Serum-Osmolalität lässt sich anhand der Gl. 1 abschätzen (Harnstoff und Glukose sind unter Normalbedingungen zu vernachlässigen).

#### Formel 1.

Berechnung der Serum-Osmolalität [mosmol/kg]

$$\text{Serum-Osmolalit{\"a}t}\ [\mathrm{mosmol}/\mathrm{kg}]=2\cdot \text{Serumnatrium}\ [\mathrm{mmol}/\mathrm{l}]+\text{Blutzucker}\ [\mathrm{mg}/\mathrm{dl}]/18+\text{Serum-Harnstoff}\ [\mathrm{mg}/\mathrm{dl}]/6$$

Veränderungen in der Serum-Osmolalität führen zu Wasserverschiebungen. Bei einer **hypotonen Hyponatriämie**

Hypotone Hyponatriämie

bewirkt die höhere Osmolalität in den Zellen gegenüber dem extrazellulären Raum eine Flüssigkeitsumverteilung in die Zelle zum Ausgleich des osmotischen Gradienten und führt auf diese Weise zur **Zellschwellung**

Zellschwellung.

Eine pathologische Serum-Natriumkonzentration ist entweder Ausdruck einer Störung der Osmoregulation oder des Volumenhaushalts.

#### Merke

Eine Änderung der Natriumkonzentration ist stets als Änderung der Natriummenge im Verhältnis zum Lösungsvolumen zu interpretieren.

#### Osmoregulation

Die Messung der Osmolalität findet im **Hypophysenhinterlappen**

Hypophysenhinterlappen

statt und regelt über die Ausschüttung von ADH, welches mehrere unabhängige Wirkungsorte aufweist, die Aufrechterhaltung der Homöostase. Bei verminderter Wasserzufuhr wird aufgrund steigender Serum-Osmolalität vermehrt ADH freigesetzt. In der Niere führt das freigesetzte ADH über die Aktivierung von V2-Rezeptoren nicht nur zum Einbau von **Aquaporinen**

Aquaporinen

im Sammelrohr, sondern auch zur Aktivierung bereits bestehender Kanäle, um eine schnelle Regulation zu ermöglichen [11]. Diese unter ADH gesteigerte Wasserrückresorption führt in der Folge zu einer Antidiurese und erhöhter Harn-Osmolalität. Bei vermehrter Wasseraufnahme wird die ADH-Sekretion unterdrückt, was in der Ausscheidung mehr „freien“ Wassers resultiert, gekennzeichnet durch geringe Osmolalität des Urins.

##### Merke

Die Serum-Natriumkonzentration ist ein relativer Wert und reflektiert nicht die absolute Natriummenge im Körper.

#### Kreislaufregulation

Für die Kreislaufregulation (Volumenregulation) gilt: Neben der Osmolalität ist das effektiv zirkulierende Volumen ein entscheidender Faktor für die Ausschüttung von ADH. Mit Abnahme des zirkulierenden Blutvolumens und damit verbunden der Gefäßwandspannung im linken Vorhof, im Karotissinus und im Aortenbogen steigt die ADH-Sekretion, um über eine vermehrte Wasserrückresorption in der Niere das fehlende Volumen zu kompensieren [12]. Von den **Barorezeptoren**

Barorezeptoren

wahrgenommen werden kann jedoch nur das im **Gefäßsystem**

Gefäßsystem

zirkulierende Volumen, nicht das Gesamtflüssigkeitsvolumen im Körper. Im Zustand der Eu- oder Hypervolämie wird die ADH-Sekretion ausschließlich anhand der Osmolalität reguliert, während bei Hypovolämie zusätzlich eine Stimulation der Sekretion durch Barorezeptoren vorliegt. Osmo- und Barorezeptoren regulieren unabhängig voneinander. Eine durch Hypovolämie getriggerte ADH-Freisetzung führt meist zu einem Abfall der Serum-Natriumkonzentration [11, 13].

##### Merke

Bei intravasaler Hypovolämie dominiert die Volumenregulation gegenüber der Osmoregulation selbst zum Preis einer sich dabei entwickelnden Hyponatriämie.

### Pathophysiologische Gesichtspunkte

#### Zellschwellung und Gegenregulation

Auf zellulärer Ebene führen Schwankungen in der Serum-Natriumkonzentration zu **Flüssigkeitsverschiebungen**

Flüssigkeitsverschiebungen

zwischen den Zellen und dem **interzellulären Raum**

Interzellulärer Raum.

Von dieser Zellschwellung sind alle Zellen im Körper betroffen, jedoch reagieren die Zellen im Gehirn empfindlicher auf Schwankungen des Zellvolumens, da eine Ausdehnung des Gehirns durch die Schädelknochen räumlich begrenzt ist. Die **zelluläre Volumenregulation**

Zelluläre Volumenregulation

versucht, dieser osmotisch bedingten Schwellung entgegenzuwirken, indem sie osmotisch wirksame Teilchen aus der Zelle transportiert. Eine schnelle Adaptation tritt innerhalb von Stunden ein und transportiert Elektrolyte aus der Zelle, jedoch reicht diese nicht, um das ursprüngliche Volumen wiederherzustellen. Im weiteren Verlauf kommt es zur langsamen Adaptation, bei welcher **organische Osmolyte**

Organische Osmolyte

aus der Zelle transportiert werden. Die **Transportkapazität**

Transportkapazität

hierfür ist allerdings deutlich geringer ausgeprägt und benötigt zwischen 24 und 48 h [6, 14]. Eine schwere Symptomatik ist somit Hinweis auf eine unvollständige zelluläre Volumenregulation und damit auf ein akutes Geschehen.

##### Merke

Die Volumenregulation der Hirnzellen führt zur Freisetzung von Osmolyten, um die Osmolalitätserniedrigung im Serum auszugleichen, und benötigt etwa 48 h.

#### Klinische Symptomatik

Generelle Symptome einer Hyponatriämie sind meist unspezifisch und umfassen **Gangunsicherheit**

Gangunsicherheit,

**Schwindel** oder leichte **Kopfschmerzen**

Kopfschmerzen

[4, 15]. Eine **akute Hyponatriämie**

Akute Hyponatriämie

manifestiert sich vordergründig mit neurologischen Symptomen als Resultat der hyponatriämischen **Enzephalopathie**

Enzephalopathie.

Zum Spektrum der Symptome gehören kognitive Beeinträchtigungen wie Konzentrationsdefizite oder Somnolenz bis hin zu Erbrechen, **Krampfanfällen**

Krampfanfällen

und schweren **Bewusstseinsstörungen**

Bewusstseinsstörungen

[6]. Der Schweregrad der Symptomatik korreliert mit der Geschwindigkeit der Entstehung der Hyponatriämie und ist somit mehr ein Ausdruck der Dynamik (Geschwindigkeit, mit der das Natrium fällt) als des absoluten Unterschieds. Eine schwere Symptomatik bei nur moderat gesenkten Serum-Natriumkonzentrationen spricht eher für eine akute Hyponatriämie (< 48 h), während bei einer profunden Hyponatriämie kombiniert mit geringen Symptomen von einem chronischen Geschehen (> 48 h/unbekannt) auszugehen ist. Bei einer **Hypervolämie**

Hypervolämie

können (zusätzlich) kardiorespiratorische Symptome wie akute **kardiale Dekompensation**

Kardiale Dekompensation

oder **hypertensive Entgleisung**

Hypertensive Entgleisung

auftreten.

##### Merke

Faustregel:

Schwere Symptomatik und moderate Hyponatriämie spricht meist für eine akute Hyponatriämie.

Leichte Symptomatik und profunde Hyponatriämie spricht meist für eine chronische Hyponatriämie.

### Ätiologie der Hyponatriämie im akutmedizinischen Setting

#### Häufige Ursachen

##### Inadäquate Antidiurese (Syndrom der inadäquaten ADH-Sekretion, SIADH).

Bei einer pathologisch **erhöhten ADH-Konzentration**

Erhöhte ADH-Konzentration

kommt es zu verminderter Wasserausscheidung bei erhöhter Harn-Osmolalität, also dem Syndrom der inadäquaten Antidiurese (Syndrom der inadäquaten ADH-Sekretion, SIADH). Die übermäßige Ausschüttung von ADH erfolgt hierbei unabhängig von einem osmotischen Stimulus. Eine pathologisch **gesteigerte Freisetzung**

Gesteigerte Freisetzung

kann in der Hypophyse (Medikamente, zentrale Stimuli wie Übelkeit oder Schmerz) oder ektopisch, an anderer Stelle, wie in der Lunge (z. B. kleinzelliges Bronchuskarzinom, schwere Pneumonie [16]) stattfinden [6]. Notfallmedizinisch sind v. a. medikamentös induzierte SIADH durch **Drogenkonsum**

Drogenkonsum

(Ecstasy, Opiate usw.) von Bedeutung. Zur Diagnosestellung einer inadäquaten Ausschüttung von ADH (SIADH) wurden essenzielle und unterstützende Kriterien erarbeitet ([17]; Tab. 2).

**Table 2:** 

Essenzielle Kriterien	Unterstützende Kriterien
Wirksame Serum-Osmolalität < 275 mosmol/kg	Serum-Harnsäure < 0,24 mmol/l (< 4 mg/dl)
Klinische Euvolämie	Serum-Harnstoff < 3,6 mmol/l (< 21,6 mg/dl)
Urin-Osmolalität > 100 mosmol/kg bei etwas verringerter wirksamer Osmolalität	Korrektur der Hyponatriämie durch Flüssigkeitsrestriktion
Urin-Natriumkonzentration > 30 mmol/l bei normaler Salz- und Wasseraufnahme	Erfolglose Korrektur der Hyponatriämie nach Infusion mit 0,9%iger Kochsalzlösung
Keine Nebennieren‑, Schilddrüsen‑, Hypophysen- oder Niereninsuffizienz	Fraktionelle Harnsäureexkretion > 12 %
Keine kürzliche Diuretikaeinnahme	Fraktionelle Natriumexkretion > 0,5 %
–	Fraktionelle Harnstoffexkretion > 55 %

##### Intravasale Hypovolämie.

Wie bereits bei der Volumenregulation beschrieben, wird die Ausschüttung von ADH auch über das effektiv zirkulierende Volumen reguliert. Eine auf diese Weise entstehende Hyponatriämie kann mit dem Bild einer **manifesten Hypovolämie**

Manifeste Hypovolämie

(z. B. Dehydrierung, diuretikavermittelte Volumendepletion, Nebennierenrindeninsuffizienz, Hypothyreose) oder **peripheren Hypervolämie**

Periphere Hypervolämie

(z. B.: Herzinsuffizienz, Leberinsuffizienz, nephrotisches Syndrom), welche sich im Auftreten von Ödemen zeigt, assoziiert sein [6, 18]. Dieser auf den ersten Blick paradox erscheinende Konstellation liegt der Umstand zugrunde, dass all diese Erkrankungen durch ein erniedrigtes zirkulierendes Volumen gekennzeichnet sind. Trotz der klinischen Interpretation einer Hypervolämie besteht aufgrund der zugrunde liegenden Erkrankung, oftmals verstärkt durch den Einsatz von Diuretika, eine intravasale Hypovolämie. Folglich nehmen die Barorezeptoren ein zu geringes intravasales Volumen wahr, und die **ADH-bedingte Flüssigkeitsrückresorption**

ADH-bedingte Flüssigkeitsrückresorption

verdünnt das im Serum verbliebene Natrium. Die Aktivierung der Barorezeptoren bewirkt auch eine **vermehrte Natriumretention**

Vermehrte Natriumretention

der Nieren, wodurch die Natriumkonzentration im Harn typischerweise erniedrigt ist, es sei denn, es besteht eine zeitgleiche Diuretikatherapie mit daraus resultierender Natriurese. **Spezielle Formen**

Spezielle Formen

einer Hypovolämie stellen die endokrinen Störungen der (akuten) Nebennierenrindeninsuffizienz oder der schweren Hypothyreose dar.

##### Merke

Eine intravasale Hypovolämie kann trotz des klinischen Eindrucks einer Hypervolämie (Ödeme) bestehen.

##### Übermäßige Flüssigkeitszufuhr/Polydipsie.

Eine Zufuhr von hypotoner Flüssigkeit reduziert die Serum-Osmolalität und aktiviert damit die Wasserdiurese [19]. Die zugeführte Flüssigkeit muss aufgrund der Funktionsweise der Niere mit einem Mindestgehalt an Elektrolyten ausgeschieden werden (Molenlast; 10 mosmol/kgKG/Tag bei ausgewogener Ernährung). Bei gesunden Nieren liegt die Grenze der maximalen Verdünnung des Urins im Bereich von 50 mosmol/l. Eine 70 kg schwere Person wäre somit in der Lage, 14 l Urin im Tag auszuscheiden, ohne Wasser zu retinieren. Ernährt sich die Person im Extremfall nur von Tee und Toast („Tea-and-Toast-Syndrom“) oder Bier („beer-drinking potomania“), so sinkt die Molenlast und das Volumen, welches ausgeschieden werden kann [20, 21, 22]. Jeder Liter über dieser Grenze kann, selbst bei absolut unterdrückter ADH-Sekretion, nicht mehr ausgeschieden werden und wird retiniert. Jenes zusätzlich im Körper verbliebene Volumen verdünnt die absolute Natriummenge im Körper und resultiert in einer niedrigeren Serum-Natriumkonzentration. Die Grenze der möglichen Ausscheidung kann jedoch auch bei regulärer Elektrolytzufuhr überschritten werden, wenn die Patient*innen (z. B. aufgrund einer psychischen Erkrankung oder übereifriger Befolgung von Hydratationsprotokollen im Rahmen einer Koloskopievorbereitung) enorme Mengen (mehrere Liter) an Wasser in kurzer Zeit zu sich nehmen. Iatrogen kann eine **Volumenüberladung**

Volumenüberladung

im Rahmen eines **TURP-Syndroms**

TURP-Syndrom

zustande kommen. Dieses zunehmend an Bedeutung verlierende Syndrom entsteht bei transurethralen Prostataresektionen (TURP) durch die Irrigation mit hypotonen Spüllösungen. Üblicherweise wird eine 1,5%ige Glycin-Lösung mit einer Gesamtosmolalität von 200 mosmol/kg verwendet. Bei längerer Operationsdauer (> 1 h) werden größere Menge absorbiert (10–30 ml/min Resektionszeit), wodurch es zu einer akuten Hyponatriämie mit Volumenüberladung kommt (Tab. 3).

**Table 3:** 

Tea-and-Toast-Syndrom
Primäre Polydipsie
Beer Potomania
Koloskopievorbereitung
Belastungsassoziierte Hyponatriämie
TURP(transurethrale Prostataresektion)-Syndrom

##### Merke

Die Niere benötigt Osmolyte, um Wasser auszuscheiden. Stehen diese nicht ausreichend zur Verfügung, verbleibt Wasser im Körper und verdünnt das Natrium.

#### Seltenere Ätiologien

##### Belastungsassoziierte Hyponatriämie („exercise-associated hyponatremia“, EAH).

Bei **Extremsportlern**

Extremsportler

(z. B. Marathonlauf) kann die Kombination aus einer übermäßigen Wasserzufuhr und dem Verlust von Natrium über den Schweiß zum Krankheitsbild einer belastungsassoziierten Hyponatriämie (EAH) mit Lungen- und **Hirnödem**

Hirnödem

führen [23, 24, 25, 26]. Das Risiko, dieses Krankheitsbild zu entwickeln, ist hierbei auch von der **individuellen Schweißproduktion**

Individuelle Schweißproduktion

abhängig (Schweißosmolalität, -natriumgehalt, -volumen) [27, 28, 29]. Zudem ist auch hier eine inadäquate, nicht vollständig unterdrückte ADH-Sekretion selbst bei normaler Serum-Osmolalität durch intensive sportliche Belastung von Relevanz [30]. Die Inzidenz der EAH hängt von der Dauer der Belastung ab. In Marathon- und Triathlonläufen wird eine Inzidenz bis zu 18 % beschrieben, während diese bei Läufen mit einer Distanz von 161 km auf bis zu 51 % ansteigen kann [31]. Insgesamt treten Symptome aufgrund einer EAH jedoch selten auf (1 % der Fälle) [32].

##### Merke

Ungünstige Kombinationen aus Osmolalität, Natriumgehalt und Schweißvolumen können bei nichtunterdrückter ADH-Sekretion bei Extremsportlern zur EAH führen.

##### Zerebrales Salzverlustsyndrom.

Pathologische Veränderungen im ZNS können die **zentrale Steuerung**

Zentrale Steuerung

der Natriumhomöostase beeinträchtigen. Patient*innen mit einer lokalen Schädigung des ZNS wie einer **Subarachnoidalblutung**

Subarachnoidalblutung

[33], einem Schädel-Hirn-Trauma [34] oder einem Schlaganfall [35] sowie hirntote Patient*innen

Hirntote Patient*innen

können ein zerebrales Salzverlustsyndrom mit Folgen für den Natriumhaushalt entwickeln. Kennzeichen für das Vorliegen ist eine erhöhte Serum-Harnstoffkonzentration bei gleichzeitig erniedrigter Serum-Harnsäurekonzentration sowie Zeichen einer Hypovolämie wie erniedrigter zentralvenöser Druck oder orthostatische Hypotension [6]. Zusätzlich liegt eine erhöhte Natriumausscheidung über 24 h vor, welche sich durch eine Urin-Natriumkonzentration ≫ 30 mmol/l und erhöhtes Harnvolumen ergibt (Tab. 4). Vordergründig scheint eine vermehrte Freisetzung von B‑Typ-natriuretischem Peptid verantwortlich zu sein, jedoch sind die pathophysiologischen Mechanismen nicht vollständig geklärt [36, 37, 38].

**Table 4:** 

Krankheitsbild	Diagnostik	
	Hyponatriämie +	
	Harn-Osmolalität	Zusätzliches Diagnosekriterium
SIADH (Syndrom der inadäquaten Antidiurese)	> 100	Harn-Natrium ≥ 30
Intravasale Hypovolämie	> 100	Harn-Natrium < 30 (nicht unter Diuretikawirkung!)
Übermäßige Flüssigkeitszufuhr	< 100	Harn-Natrium variabel
Zerebrales Salzverlustsyndrom	> 300	Harn-Natrium ≥ 30 (24 h Natriumausscheidung erhöht)
Belastungsassoziierte Hyponatriämie	variabel	Extreme körperliche Belastung

##### Merke

Pathologische Veränderungen im ZNS können Auswirkungen auf den Natriumhaushalt haben.

## Handlungsalgorithmus bei Hyponatriämie

Zentrale Aspekte der Behandlung einer Hyponatriämie sind die Interpretation der Laborwerte sowie die Einschätzung der klinischen Symptomatik. Auf die einzelnen Schritte wird im Folgenden eingegangen.

### Schritt 1: Hypoosmolalität verifizieren

Zu Beginn des Behandlungsalgorithmus gilt es, den abnormen Laborbefund einer hypotonen Hyponatriämie zu verifizieren und eine Pseudohyponatriämie auszuschließen (Gl. 1). Falls das Natrium mittels **Blutgasanalyse**

Blutgasanalyse

bestimmt wurde, entfällt dieser Schritt. Auszuschließen wäre zudem das Vorliegen **exogener Osmolyte**

Exogene Osmolyte

(z. B. Hyperglykämie). Eine durch eine **Hyperglykämie**

Hyperglykämie

entstehende Umverteilung von freier Flüssigkeit führt zwar ebenso zu einer Hyponatriämie, jedoch aufgrund der osmotisch wirksamen Glukose zu keiner Hypoosmolalität [39]. Die resultierende isotone/**hypertone Hyponatriämie**

Hypertone Hyponatriämie

bedarf keiner Therapie. Die korrigierte Serum-Natriumkonzentrationen kann mithilfe der Gl. 2 errechnet werden, um eine durch Hyperglykämie entstandene Hyponatriämie auszuschließen. Liegt eine **hypotone Hyponatriämie**

Hypotone Hyponatriämie

vor, folgt Schritt 2.

#### Formel 2.

Korrigierte Serum-Natriumkonzentration bei Hyperglykämie

$$\text{Korr. Serum}\ (\mathrm{Na}^{+})= \text{gemessenes}\,\mathrm{Na}^{+} + 2{,}4 \cdot \frac{\text{Glukose}\,\left(\mathrm{mg}/\mathrm{dl}\right)-100\,\left(\mathrm{mg}/\mathrm{dl}\right)} {100\,\left(\mathrm{mg}/\mathrm{dl}\right)}$$

$$\text{Korr. Serum}\ (\mathrm{Na}^{+})=\text{gemessenes}\,\mathrm{Na}^{+}+2{,}4 \cdot \frac{\text{Glukose}\,\left(\mathrm{mmol}/\mathrm{l}\right)-5{,}5\,\left(\mathrm{mmol}/\mathrm{l}\right)}{5{,}5\,\left(\mathrm{mmol}/\mathrm{l}\right)}$$

### Schritt 2: Einschätzung der Klinik und Therapieinitiierung

Beim Vorliegen einer **mittelschweren oder schweren Symptomatik**

**Figure 2:**
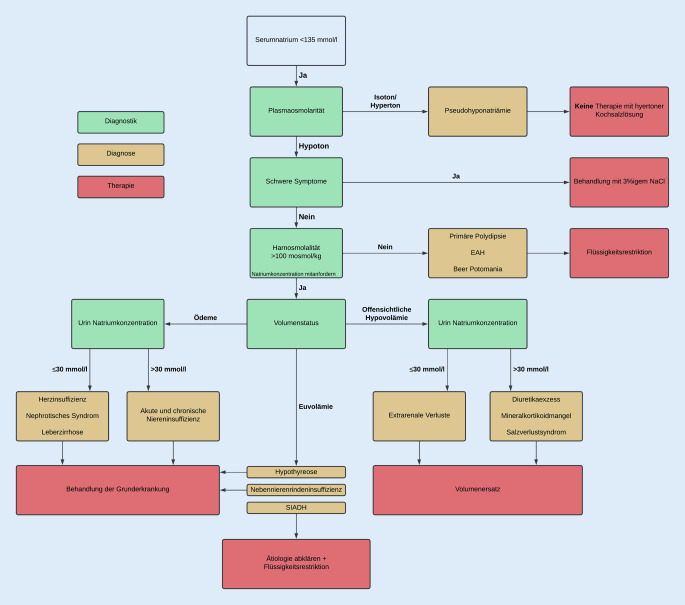

Mittelschwere oder schwere Symptomatik

(Tab. 1) steht die rasche Therapieinitiierung im Vordergrund. Andernfalls kann direkt mit Schritt 3 (weiterführende Diagnostik) weitergemacht werden. Für die weiterführende Diagnostik sollte auch bei mittelschwerer oder schwerer Symptomatik vor Therapieinitiierung eine Harnprobe gewonnen werden (Abb. 2).

Bei **Hyper- und Euvolämie**

Hyper- und Euvolämie

können 150 ml einer 3%igen NaCl-Lösung über 20 min ohne Bedenken verabreicht werden. Dies kann im 20-min-Intervall mit bis zu insgesamt 4 Infusionen wiederholt werden, um eine Steigerung von 6 mmol/l in 6 h zu erreichen: **6‑by-6-Regel**

6‑by-6-Regel.

Bei Übergewichtigen oder schlanken Personen empfiehlt sich anstatt der 150 ml eine **gewichtsbezogene Verabreichung**

Gewichtsbezogene Verabreichung

von 2 ml/kgKG, da bei fixen Dosierungen in Abhängigkeit vom Körpergewicht sowohl Über- als auch Unterkorrekturen beschrieben sind [40].

#### Merke

Als Faustregel kann Folgendes angenommen werden:

1 ml/kgKG einer 3%igen NaCl-Lösung hebt die Serum-Natriumkonzentration etwa um 1 mmol/l.

Kommt es nach der Initialtherapie zur **Besserung**

Besserung

der Symptome, sollte die Therapie mit 3 % NaCl gestoppt und die Serum-Natriumkonzentration nach 6 h, 12 h und ab **Stabilisierung**

Stabilisierung

täglich gemessen werden.

Ist keine Besserung zu beobachten, sollte die Therapie mit 3 % NaCl fortgeführt werden bis entweder 130 mmol/l Serum-Natriumkonzentration *oder* eine Steigerung von insgesamt 10 mmol/l in 24 h erreicht worden ist *oder* sich die **Symptome gebessert**

Symptome gebessert

haben [6]. Die empfohlene Zunahme der Serum-Natriumkonzentration beträgt 1 mmol/l pro Stunde. Der hierfür benötigte Natriumbedarf kann mithilfe der Adrogue-Madias-Formel abgeschätzt werden. Bei ausbleibender Besserung der Symptome sollte zudem eine andere Ursache mittels einer **bildgebenden Diagnostik**

bildgebende Diagnostik

ausgeschlossen werden.

#### Formel 3.

Adrogue-Madias-Formel

$$\mathrm{Na}^{+}\,\text{Korrektur pro Liter}=\frac{\mathrm{Na}^{+}\text{Konzentration in Infusion}\,\left(\mathrm{mmol/l}\right)-\mathrm{Na}^{+}\text{Konzentration im Serum}\,\left(\mathrm{mmol/l}\right)}{\text{Gesamtk{\"o}rperwasser}^{*}+1}$$

$$\frac{\text{gew{\"u}nschte Korrektur des } \mathrm{Na}^{+}}{\mathrm{Na}^{+}\,\text{Korrektur pro Liter}}=\text{Liter der Infusionsl{\"o}sung}$$

* Gesamtkörperwasser = KG × Faktor.

Der Faktor entspricht je nach Alter und Körperzusammensetzung:

- 0,6 bei Kindern und bei Männern < 70 Jahren
- 0,5 bei Frauen < 70 Jahren und bei Männern ≥ 70 Jahren,
- 0,45 bei Frauen ≥ 70 Jahren

1000 ml **0,9** **%** NaCl beinhalten 9 g entsprechend **154** **mmol** Na (308 mosmol/kg)

1000 ml **3** **%** NaCl beinhalten 30 g entsprechend **513** **mmol** Na (1026 mosmol/kg)

1000 ml **5** **%** NaCl beinhalten 50 g entsprechend **855** **mmol** Na (1710 mosmol/kg)

Der reale Anstieg des Serum-Natrium hängt neben dem Körpergewicht von verschieden Faktoren wie Harn-Osmolalität und Diurese ab. Liegt die Harn-Osmolalität über der Osmolalität der verabreichten Flüssigkeit, kommt es zu einer Wasserretention und damit zu einem weiteren Absinken der Serum-Natriumkonzentration. Während bei einer 3%igen NaCl-Lösung die Osmolalität praktisch immer über der Harn-Osmolalität liegt, kann die Osmolalität einer 0,9%igen NaCl-Lösung darunterliegen. Besondere Beachtung kommt dem Auftreten von **Polyurie**

Polyurie

unter Behandlung zu. Hier kann es zu einem unerwartet raschen Anstieg des Serum-Natriums kommen.

Klinisch manifest hypovoläme Patient*innen stellen in der Behandlung der hypotonen Hyponatriämie eine Sonderform dar. Hier gilt es, die Hypovolämie (zusätzlich) mittels **isotonen Kristalloiden**

Isotone Kristalloide,

bevorzugt 0,9 % NaCl (0,5–1 ml/kg/h), und somit gleichzeitig die übermäßige ADH-Ausschüttung und die daraus resultierende Hyponatriämie zu behandeln.

#### Merke

Liegt die Harn-Osmolalität über der Osmolalität der verabreichten Flüssigkeit, kommt es zu einer Wasserretention.

### Schritt 3: Weiterführende Diagnostik und Therapie

Im Fall einer fehlenden klinischen Manifestation oder bei leichten Symptomen sollten alle Infusionen und die Gabe potenziell SIADH auslösender Medikamente (Tab. 5), falls möglich, beendet und parallel mit der Diagnostik fortgefahren werden.

**Table 5:** 

Zentral wirksam	Peripher wirksam
*Antidepressiva*	*ADH-Analoga*
Selektive Serotonin-Wiederaufnahme-Hemmer (SSRI)	Desmopressin
Trizyklische Antidepressiva	Oxytozin
Monoaminoxidasehemmer (MAOI)	Terlipressin
Venlafaxin	Vasopressin
*Antiepileptika*	*Thiaziddiuretika*
Carbamazepin	Hydrochlorothiazid
Oxcarbazepin	*Amiodaron*
Valproinsäure	*Protonenpumpenhemmer (PPI)*
Lamotrigin	*Monoklonale Antikörper*
*Antipsychotika*	*Clofibrat*
Phenothiazine	*Levamisol*
Butyrophenon	*Interferon*
*Drogen*	*Nichtsteroidale Antirheumatika (NSAR)*
Opiate	
MDMA (3,4-Methylendioxymethylamphetamin, Ecstasy)	
Nikotin	
*Chemotherapeutika*	
Vincaalkaloide	
Platine	
Ifosfamid	
Melphalan	
Cyclophosphamid	
Methothrexat	
Pentostatin	

#### Urindiagnostik.

Die Harnanalytik erfolgt idealerweise vor Einleitung der Therapiemaßnahmen (am besten zeitgleich mit der Blutprobe). In der Harnprobe sollten sowohl die **Harn-Osmolalität**

Harn-Osmolalität

als auch die **Natriumkonzentration**

Natriumkonzentration

(falls möglich, idealerweise auch die Harnsäure und das Kreatinin im Harn) bestimmt werden. Ist die Harn-Osmolalität sehr niedrig (< 100 mosmol/kg – absolute ADH-Unterdrückung), ist eine zu große absolute/relative Flüssigkeitsaufnahme (z. B. Polydipsie) die Ursache der Hyponatriämie.

#### Volumenstatus.

Als weiterer diagnostischer Aspekt ist nach der Urindiagnostik der Volumenstatus der Patient*innen zu beurteilen (Blutdruck, Puls, **Orthostasereaktion**

Orthostasereaktion,

Halsvenen). Sowohl die Sensitivität als auch die Spezifität der klinischen Untersuchung in Hinblick auf den Volumenstatus ist jedoch gering [41, 42]. Eine periphere Hypervolämie kann durch Volumenüberladung entstehen (primär) oder sich sekundär als Folge von Krankheiten manifestieren. Liegt eine sekundäre Hypervolämie vor, ist dennoch an eine mögliche intravasale Hypovolämie zu denken. Sowohl bei den peripher **hypervolämen**

Hypervoläm

als auch bei den **hypovolämen**

Hypovoläm

Patient*innen ist die zuvor bereits veranlasste Messung der Natriumkonzentration im Urin zur weiteren Differenzierung hilfreich. Ein Natrium < 30 mmol/l lässt auf eine renale Minderperfusion und damit eine effektive Hypovolämie schließen. Bei nicht verwertbaren Natriumkonzentrationen im Urin aufgrund von Diuretikaeinnahme sollte die fraktionelle **Harnsäureexkretionsrate**

Harnsäureexkretionsrate

(< oder > 12 %) mithilfe des Kreatinins bestimmt und zur Unterscheidung genutzt werden. Eine Harnsäureexkretionsrate von < 12 % spricht für ein erniedrigtes effektiv zirkulierendes Blutvolumen.

#### Merke

Die Harnanalytik erfolgt idealerweise vor Einleitung der Therapiemaßnahmen (am besten zeitgleich mit der Blutprobe).

#### Formel 4.

Formel zur Berechnung der fraktionellen Harnsäureexkretion in Prozent

$$\text{Fraktionelle Exkretion}\left(\text{Harns{\"a}ure}\right)=\frac{\text{Harns{\"a}ure}\,\left(\text{Urin}\right)\cdot\text{Kreatinin}\,\left(\text{Serum}\right)}{\text{Kreatinin}\,\left(\text{Urin}\right)\cdot\text{Harns{\"a}ure}\,\left(\text{Serum}\right)}\times 100$$

#### Behandlung zugrunde liegender Krankheitsbilder

##### SIADH.

Primäres Ziel sind Identifizierung und Behebung der auslösenden Ursache (z. B. Absetzen psychotroper Substanzen), verbunden mit einer **Flüssigkeitsrestriktion**

Flüssigkeitsrestriktion.

Nach Beheben der Ursache kommt es meist innerhalb von 24–48 h zu einer Normalisierung der ADH-Freisetzung, wobei auf eine zu schnelle Korrektur geachtet werden sollte (s. Fallbeispiel). Erst wenn diese Maßnahme nicht erfolgreich ist, sollte auf eine **tägliche Gabe**

Tägliche Gabe

von **Harnstoff**

Harnstoff

0,25–0,50 g/kg oder die Verabreichung von **Schleifendiuretika**

Schleifendiuretika

in Kombination mit **oralem NaCl**

Orales NaCl

umgestiegen werden. Nur bei gesicherter Diagnose eines SIADH und unter engmaschiger Serum-Natriumkontrolle (6-h-Intervall in den ersten 48 h) kann als letzter Schritt die Verabreichung eines Vasopressin-Rezeptor-Antagonisten, z. B. **Tolvaptan**

Tolvaptan,

erfolgen. Cave: Nach Gabe eines Vasopressin-Rezeptor-Antagonisten sind rasche und hohe Anstiege der Serum-Natriumkonzentration beschrieben [43, 44]. Eine Dosis von 7,5 mg statt der empfohlenen 15 mg hat sich als effektiv und sicher erwiesen [45, 46].

##### Intravasale Hypovolämie.

Manifeste Hypovolämie bedarf zusätzlich eines Volumenersatzes mit 0,5–1 ml/kg/h von 0,9 % NaCl (oder **balancierten Kristalloiden**

Balancierte Kristalloide)

[6], um die erhöhte ADH-Sekretion zu reduzieren. Bei ödematösen Zustandsbildern (d. h. Herzinsuffizienz, Leberzirrhose, nephrotisches Syndrom) besteht die Behandlung in der Therapie der Grunderkrankung.

##### Übermäßige Zufuhr von hypotoner Flüssigkeit.

Therapeutisch sollte hier primär eine Flüssigkeitsrestriktion angestrebt und die Grunderkrankung therapiert werden.

##### Belastungsassoziierte Hyponatriämie.

Die belastungsassoziierte Hyponatriämie ist **selbstlimitierend**

Selbstlimitierend,

und nur beim Auftreten von Symptomen sollte die Verabreichung einer hypertonen Lösung erwogen werden.

##### Zerebrales Salzverlustsyndrom.

Die ausgeprägte Natriurese führt zu einem extrazellulären Flüssigkeitsverlust und zur Hypovolämie. Es wird empfohlen, das zerebrale Salzverlustsyndrom mit 0,9 % NaCl oder einer balancierten kristalloiden Lösung (je nach Harn-Osmolalität) zu behandeln. Bei einer Harn-Osmolalität > 300 mosmol/kg ist **hypertone Kochsalzlösung**

Hypertone Kochsalzlösung

kontinuierlich bis zur Normalisierung des Serum-Natriums zu verabreichen.

### Schritt 4: engmaschiges Monitoring des Natriumverlaufs und Vermeidung von Komplikationen

Unter engmaschigen Kontrollen ist die Messung der Serum-Natriumkonzentration nach 6 und 12 h bei Besserung der Symptome nach einem initialen Anstieg um 6 mmol/l zu verstehen. Solange mit einer 3%igen hypertonen NaCl-Lösung therapiert wird, ist eine Messung alle 4 h empfohlen. Um einen zu raschen Anstieg der Serum-Natriumkonzentration durch Wiedereinsetzen der Diurese nicht zu übersehen, sollte die **Harnausscheidung quantitativ**

Harnausscheidung quantitativ

erfasst werden (**Cave**: Polyurie).

#### Osmotisches Demyelinisierungssyndrom.

**Figure 3:**
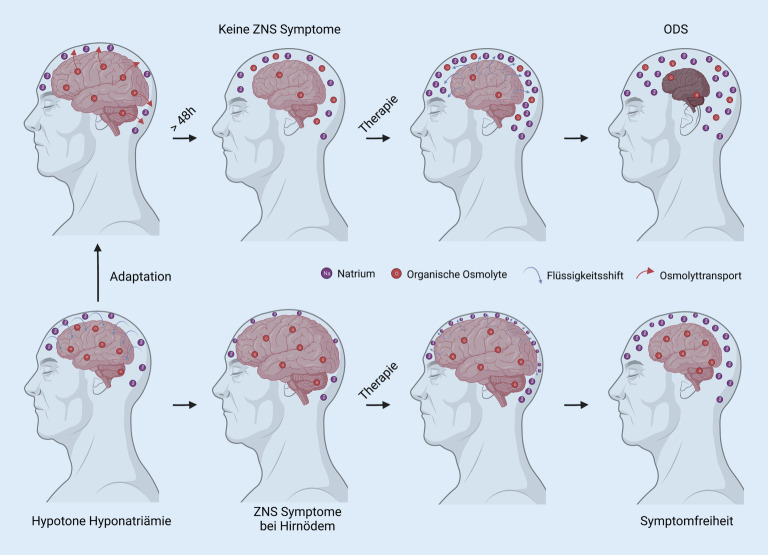

Eine zu schnelle Korrektur der Serum-Natriumkonzentration ist Ursache des osmotischen Demyelinisierungssyndroms (Abb. 3), welches bei 0,28–0,5 % der Hyponatriämien auftritt [47, 48, 49]. Entscheidend ist beim Demyelinisierungssyndrom der zeitliche Zusammenhang bei der Entstehung der Hyponatriämie. Je langsamer die Serum-Natriumkonzentration fällt, desto mehr Zeit haben die Zellen, sich zu adaptieren. Im Rahmen der Adaptation wird die **intrazelluläre Osmolalität**

Intrazelluläre Osmolalität

gesenkt und hypoton gegenüber dem Normzustand vor Adaptation. Haben die **Hirnzellen**

Hirnzellen

in der Therapie nicht genug Zeit zu einer Umkehr der Adaptation, kommt es zum Demyelinisierungssyndrom. Die Symptome des Demyelinisierungssyndroms [50, 51] reichen von einer Schwäche in den Extremitäten bis zum **Atemstillstand**

Atemstillstand

[49, 52]. Risikofaktoren für ein osmotisches Demyelinisierungssyndrom sind in der Tab. 6 angeführt.

**Table 6:** 

Alkoholerkrankung
Lebererkrankung
Hypokaliämie
Mangelernährung
Profunde Hyponatriämie

Befinden sich die Zellen bereits im adaptierten Zustand, muss die notwendige Korrektur des Serum-Natriums langsam erfolgen, während bei Auftreten innerhalb von 48 h, also bei akuter Hyponatriämie, nötigenfalls ein schnellerer Anstieg akzeptiert werden kann. Bei **geringer Symptomatik**

Geringe Symptomatik

und Unklarheit über den Beginn oder > 48 h sollte den **Korrekturgrenzen**

Korrekturgrenzen

besondere Beachtung geschenkt werden. Ein Demyelinisierungssyndrom tritt im Großteil der Fälle auf, wenn die Korrekturgrenzen nicht eingehalten werden, ist jedoch auch bei deren Einhaltung möglich [54, 55, 56].

#### Merke

Als Grenze ist ein maximaler Anstieg um 10 mmol/l innerhalb der ersten 24 h und 8 mmol/l in den darauffolgenden 24 h (bis eine Serum-Natriumkonzentration von 130 mmol/l erreicht wird) zu beachten. Um die Grenzen einzuhalten, empfiehlt sich eine Messung nach jeder Infusion.

#### Maßnahmen bei Überkorrektur.

Eine Überkorrektur liegt vor, wenn die Korrekturgrenzen nicht eingehalten wurden. Im ersten Schritt muss die Behandlung, welche zur Überkorrektur geführt hat, beendet und eine **elektrolytfreie Infusion**

Elektrolytfreie Infusion

begonnen werden. Empfohlen werden hierbei 10 ml/kgKG einer 5%igen **Glukoselösung**

Glukoselösung

über eine Stunde. Reicht dies nicht aus, kann man 2 µg **Desmopressin**

Desmopressin

alle 8 h verabreichen [6].

#### Maßnahmen bei Auftreten einer Polyurie.

Als Folge der Flüssigkeitsgabe bei hypovolämen Patient*innen oder nach Absetzen der Medikamente kann die durch ADH-Stimulation vermittelte Antidiurese sistieren. Daher ist auf die Urinproduktion (> 100 ml/h) zu achten. Mit der **wiedereinsetzenden Diurese**

Wiedereinsetzende Diurese

geht ein schnellerer Anstieg der Serum-Natriumkonzentration einher. In diesem Fall ist eine Kontrolle alle 2 h zu empfehlen und ggf. die beschriebenen Maßnahmen bei Überkorrektur zu ergreifen.

#### Korrektur einer gleichzeitig bestehenden Hypokaliämie.

Störungen der Elektrolyte treten häufig gemeinsam auf. Ist neben der Korrektur von Natrium auch eine Substitution von Kalium notwendig, so bedarf dies besonderer Aufmerksamkeit. Der Hypokaliämie ist bei der **Elektrolytsubstitution**

Elektrolytsubstitution

der Vorrang zu geben, und diese steigert bereits die Serum-Natriumkonzentration. Bei gleichzeitiger Verabreichung kommt es zu einem schnelleren Anstieg der Serum-Natriumkonzentration mit der Gefahr einer Überkorrektur.

#### Formel 5.

Vereinfachte Edelman Formel

$$\Delta \,\text{Serum}\left(\mathrm{Na}^{+}\right)=\frac{\left(\mathrm{Na}^{+}\right)+\left(\mathrm{K}^{+}\right)}{\text{Gesamtk{\"o}rperwasser}^{*}}$$

* Gesamtkörperwasser = KG × Faktor.

Der Faktor entspricht je nach Alter und Körperzusammensetzung:

- 0,6 bei Kindern und bei Männern < 70 Jahren
- 0,5 bei Frauen < 70 Jahren und bei Männern ≥ 70 Jahren,
- 0,45 bei Frauen ≥ 70 Jahren

#### Formel 6.

Osmolalität einer Lösung mit Kaliumchloridzusatz

Osmolalität einer 0,9%igen NaCl-Lösung mit dem Zusatz von 40 mEq (40mmol) Kaliumchlorid:

$$0{,}9\,{\%}\, \mathrm{NaCl}\,1000\,\mathrm{ml}+40\,\mathrm{mEq}\,\mathrm{KCl}=308\,\mathrm{mosmol}+80\,\mathrm{mosmol}=388\,\mathrm{mosmol}$$

#### Merke

Der Hypokaliämie ist bei der Elektrolytsubstitution der Vorrang zu geben, und diese steigert bereits die Serum-Natriumkonzentration.

## Fazit für die Praxis

- Natrium ist als Hauptdeterminante entscheidend für die Osmolalität im Extrazellulärraum verantwortlich.
- Die hypoosmotische Hyponatriämie ist ein häufiges Krankheitsbild, welches im Akutfall einer raschen Therapieinitiierung bedarf.
- Für die akute, hypotone Hyponatriämie gilt die Infusion mit 2 ml/kgKG einer 3%igen NaCl-Lösung über 20 min als Therapie der Wahl.
- Dies kann unter Einhaltung bestimmter Grenzen bis zur Besserung der Symptomatik mehrfach wiederholt werden.
- Die engmaschige Überwachung des Serum-Natriums unter Beobachtung von klinischen Veränderungen zur Vermeidung von Überkorrektur ist dabei essenziell.
- Die Pathophysiologie ist vielschichtig und bedarf einer strukturierten Diagnostik zur Ursachenermittlung.
- Um der Gefahr des Demyelinisierungssyndroms vorzugbeugen, ist die Einhaltung von Korrekturgrenzen angeraten.

## CME-Fragebogen

Ein Patient klagt in der Notaufnahme über Kopfschmerzen seit 4 Tagen. Die neurologische Untersuchung durch den Facharzt ergibt einen dem Alter entsprechenden Normbefund des Patienten. Eine bei Aufnahme durchgeführte Routinelaboruntersuchung weist eine Natriumkonzentration von 131 mmol/l aus. Welche Aussage stimmt am ehesten mit der Definition der europäischen Leitlinie zur Hyponatriämie überein?

Leichte Hyponatriämie mit therapeutisch optionaler Serumnatrium Normalisierung mit hypertoner Kochsalzlösung

Leichte Hyponatriämie ohne akute Behandlungsbedürftigkeit mit hypertoner Kochsalzlösung

Moderate Hyponatriämie ohne akute Behandlungsbedürftigkeit mit hypertoner Kochsalzlösung

Profunde Hyponatriämie ohne akute Behandlungsbedürftigkeit mit hypertoner Kochsalzlösung

Leichte Hyponatriämie ohne akute Behandlungsbedürftigkeit mit hypoosmotischer Kochsalzlösung

Die chronische Hyponatriämie ist definiert durch die Entwicklung über eine Dauer von?

> 12 h

≥ 48 h/unbekannt

< 48 h

> 24 h

> 72 h

Welches ist ein essenzielles Diagnosekriterium des Syndroms der inadäquaten ADH-Sekretion (SIADH)?

Serum-Harnsäure < 0,24 mmol/l (< 4 mg/dl)

Erfolglose Korrektur der Hyponatriämie nach 0,9%iger Kochsalzinfusion

Fraktionelle Harnsäureexkretion > 12 %

Wirksame Serum-Osmolalität < 275 mosmol/kg

Korrektur der Hyponatriämie durch Flüssigkeitsrestriktion

Welcher Parameter spielt in der Adrogue-Madias-Formel eine Rolle?

Gesamtkörperwasser

Körpergröße

Body Mass Index (BMI)

Körperfettanteil

Hautfarbe

Welches Krankheitsbild ist *nicht* ursächlich für Laborartefakte bei Hyponatriämie?

Hyperglykämie

M. Waldenström

Hypertriglyzeridämie

Multiples Myelom

Urämie

Sie werden am Nachmittag zu einem stationären Patienten (75 kg Körpergewicht) gerufen. Die Pflege berichtet, dass der Patient am Vormittag mehrmals erbrochen hat und nun eine tiefe Schläfrigkeit aufweist. Die stationäre Aufnahme erfolgte aufgrund einer komplexen Fraktur der Hand, sonst sei er gesund und nimmt keine Dauermedikation ein. Das Natrium im Serum war bei Aufnahme (Vortag) im Normbereich, auf ein Labor am Morgen wurde verzichtet. Sie führen eine arterielle Punktion mit anschließender Blutgasanalyse durch. Hierbei zeigt sich ein Natriumwert von 124 mmol/l sowie die Glukose im Normbereich. Wie gehen Sie in dieser Situation weiter vor, um die Hyponatriämie initial zu therapieren?

Sie infundieren 75 ml (1 ml/kgKG) einer 5,85%igen Natrium-Chlorid(NaCl)-Lösung über 20 min.

Sie infundieren 500 ml (6–7 ml/kgKG) einer 3%igen Natrium-Chlorid(NaCl)-Lösung über 20 min.

Sie infundieren 150 ml (2 ml/kgKG) einer 3%igen Natrium-Chlorid(NaCl)-Lösung über 20 min.

Sie entscheiden sich gegen eine Na-Substitution und ordnen eine Laborkontrolle für den Folgetag an.

Sie infundieren 150 ml (2 ml/kgKG) einer 0,9%igen Natrium-Chlorid(NaCl)-Lösung über 24 h.

Welche Diagnose sollte mit Volumenersatz behandelt werden?

Primäre Polydipsie

Syndrom der inadäquaten ADH-Sekretion (SIADH)

Beer Potomania

Salzverlustsyndrom

Nebennierenrindeninsuffizienz

Eine 80-jährige Patientin (104 kg) wird aus einem peripheren Krankenhaus ohne Neurologie an das Zentrum überstellt. Die primäre stationäre Aufnahme erfolgte aufgrund der operativen Versorgung einer Schenkelhalsfraktur. Grund für die Transferierung ist eine zunehmende neurologische Verschlechterung seit der Operation vor 2 Wochen. Im Labor zeigt sich neben einem ansonsten altersentsprechenden Befund eine profunde Hyponatriämie (124 mmol/l). Die Patientin ist schläfrig, aber ansprechbar und antwortet auf Fragen. Ein akut neurologisches Geschehen wird mittels Bildgebung ausgeschlossen. Klinisch präsentiert sich die Patientin stark exsikkiert. Wie gehen Sie vor?

Sie stellen die Diagnose einer Exsikkose und verabreichen 0,5–1 ml/kg/h einer elektrolytfreien 5%igen Glukoselösung.

Sie stellen die Diagnose einer Hyponatriämie und verabreichen 0,5–1 ml/kg/h von 3%igem NaCl, um die Hyponatriämie zu behandeln.

Sie stellen die Diagnose einer Exsikkose und verabreichen 0,5–1 ml/kg/h einer 0,9%igen NaCl-Lösung und kontrollieren die Urinausscheidung sowie -osmolalität.

Sie stellen die Diagnose eines postoperativen Delirs und konsultieren einen Neurologen.

Sie stellen die Diagnose einer Exsikkose und verabreichen 10–15 ml/kg/h einer 0,9%igen NaCl-Lösung und kontrollieren die Urinausscheidung sowie Osmolalität.

Eine 93-jährige Patientin (73 kg) wird vom Notarzt bei Verwirrtheit seit 3 Tagen zugewiesen. Laut Ehemann kam es in den vergangenen Tagen zu einer zunehmenden Verwirrtheit, Schläfrigkeit und Übelkeit. Vor 14 Tagen wurde ihr vom Hausarzt wegen Schlafstörung ein neues Medikament verabreicht. In der Notaufnahme präsentiert sich die Patientin durchgehend ansprechbar, jedoch sehr müde. Laborchemisch zeigt sich eine ausgeprägte hypoosmolare Hyponatriämie (Na^+^ 107 mmol/l). Die übrigen Laborbefunde sind unauffällig und dem Alter der Patientin entsprechend. Die Harn-Osmolalität beträgt 350 mosmol/kg. Die Vitalparameter sind mit RR 145/85 und Herzfrequenz 75/min im Normbereich. Sie möchten die Serum-Natriumkonzentration um 6 mmol in 6 h steigern. Wie gehen Sie weiter vor?

Sie verabreichen einen errechneten Bedarf von gerundet 500 ml 3%iger NaCl-Lösung, um die Serum-Natriumkonzentration um 6 mmol über 6 h zu steigern.

Sie verabreichen einen errechneten Bedarf von gerundet 900 ml 3%iger NaCl-Lösung, um die Serum-Natriumkonzentration um 6 mmol/l über 6 h zu steigern.

Sie verabreichen einen errechneten Bedarf von gerundet 4900 ml 0,9%iger NaCl-Lösung, um die Serum-Natriumkonzentration um 6 mmol/l über 6 h zu steigern.

Sie verabreichen einen errechneten Bedarf von gerundet 500 ml 0,9%iger NaCl-Lösung, um die Serum-Natriumkonzentration um 6 mmol/l über 6 h zu steigern.

Sie verordnen 7,5 mg Tolvaptan und kontrollieren das Serum-Natrium 6 h später.

Sie bemerken bei der Therapie einer akuten Hyponatriämie einen Anstieg des Serumnatriums um 18 mmol/l in den ersten 24 h. Sie wollen sofortige Maßnahmen zu Senkung der Serum-Natriumkonzentration einleiten. Welches Vorgehen ist dazu empfohlen?

Verabreichen eines Schleifendiuretikums

Verabreichen balancierter Lösungen

Verabreichen von 10 ml/kgKG einer 5%igen Glukoselösung über 1 h

Verabreichen kolloider Lösungen

Verabreichen isotoner Kochsalzlösungen
