# Erratum zu: Hyponatriämie: Ätiologie, Diagnostik und Akuttherapie

**DOI:** 10.1007/s00063-023-01084-x

**Published:** 2023-11-23

**Authors:** Fabian Perschinka, Paul Köglberger, Sebastian J. Klein, Michael Joannidis

**Affiliations:** 1 Gemeinsame Einrichtung Internistische Intensiv- und Notfallmedizin, Department für Innere Medizin, Medizinische Universität Innsbruck, Anichstraße 35, 6020 Innsbruck, Österreich; 2 Institut für Anästhesiologie und Intensivmedizin, Klinikum Wels, Grieskirchnerstraße 42, 4600 Wels, Österreich

**Erratum zu:**

**Med Klin Intensivmed Notfmed 2023**

10.1007/s00063-023-01049-0

In der ursprünglichen Version des Artikels war ein Merksatz auf Seite 513 nicht korrekt. Er lautete:

## Merke

Als Grenze ist ein maximaler Anstieg um 10 mmol/l innerhalb der ersten 24 h und 8 mmol/l in den darauffolgenden 24 h (bis eine Osmolalität von 130 mosmol/kg erreicht wird) zu beachten. Um die Grenzen einzuhalten, empfiehlt sich eine Messung nach jeder Infusion.

Bitte beachten Sie die korrigierte Version:

## Merke

Als Grenze ist ein maximaler Anstieg um 10 mmol/l innerhalb der ersten 24 h und 8 mmol/l in den darauffolgenden 24 h (bis eine Serum-Natriumkonzentration von 130 mmol/l erreicht wird) zu beachten. Um die Grenzen einzuhalten, empfiehlt sich eine Messung nach jeder Infusion.

Der ursprüngliche Beitrag wurde korrigiert.

